# Evaluation of a multi-marker immunomagnetic enrichment assay for the quantification of circulating melanoma cells

**DOI:** 10.1186/1479-5876-10-192

**Published:** 2012-09-15

**Authors:** James B Freeman, Elin S Gray, Michael Millward, Robert Pearce, Melanie Ziman

**Affiliations:** 1 School of Medical Sciences, Edith Cowan University, Perth, WA, Australia; 2 Department of Medicine, University of Western Australia, Crawley, Australia; 3 School of Pathology and Laboratory Medicine, University of Western Australia, Crawley, Australia; 4 Director ECU Melanoma Research Foundation, Edith Cowan University (ECU), 270 Joondalup Drive, Joondalup, Perth, 6027, WA, Australia

**Keywords:** Circulating tumour cells, Melanoma, Immunomagnetic enrichment, Multi-marker

## Abstract

**Background:**

Circulating melanoma cells (CMCs) are thought to be valuable in improving measures of prognosis in melanoma patients and may be a useful marker of residual disease to identify non-metastatic patients requiring adjuvant therapy. We investigated whether immunomagnetic enrichment targeting multiple markers allows more efficient enrichment of CMCs from patient peripheral blood than targeting a single marker. Furthermore, we aimed to determine whether the number of CMCs in patient blood was associated with disease stage.

**Methods:**

We captured CMCs by targeting the melanoma associated markers MCSP and MCAM as well as the melanoma stem cell markers ABCB5 and CD271, both individually and in combination, by immunomagnetic enrichment. CMCs were enriched and quantified from the peripheral blood of 10 non-metastatic and 13 metastatic melanoma patients.

**Results:**

Targeting all markers in combination resulted in the enrichment of more CMCs than when any individual marker was targeted (p < 0.001-0.028). Furthermore, when a combination of markers was targeted, a greater number of CMCs were enriched in metastatic patients compared with non-metastatic patients (p = 0.007).

**Conclusions:**

Our results demonstrated that a combination of markers should be targeted for optimal isolation of CMCs. In addition, there are significantly more CMCs in metastatic patients compared with non-metastatic patients and therefore quantification of CMCs may prove to be a useful marker of disease progression.

## Background

Melanoma is an aggressive and drug resistant skin cancer which is responsible for 80% of skin cancer related deaths [1]. Current prognostic techniques are inadequate for disease management as many patients considered clinically disease free following primary tumour resection later develop metastases. This is highlighted by the 10 year survival rate for non-metastatic patients, which ranges from 39% to 93%, depending on the primary tumour thickness, mitotic rate and presence of ulceration [2]. There is no measure of residual disease in early stage patients post surgery and those requiring treatment are consequently unable to be identified. More sensitive measures need to be developed in order to more accurately stage patients and assist identification of early stage patients at risk of developing metastatic disease [3-5].

It is thought that the number of circulating melanoma cells (CMCs) in patient peripheral blood may be a useful prognostic marker [6-8]. The presence of circulating tumour cells is correlated with prognosis in breast, prostate and colorectal cancer patients [9-18]. CMCs can be detected by reverse transcription polymerase chain reaction (RT-PCR) and results have shown detection of melanoma markers correlates with poor prognosis [1,3,19-22]. Furthermore, RT-PCR has demonstrated expression of melanoma markers in peripheral blood of early stage patients with no clinical evidence of metastasis, suggesting CMCs are present in all disease stages [3,20,21]. The use of RT-PCR does not allow CMCs to be quantified, however, there has been promise shown by immunomagnetic enrichment, where magnetic particles conjugated to antibodies specific for melanoma antigens are used to isolate CMCs from patient blood [8,23-26].

Melanoma chondroitin sulfate proteoglycan (MCSP) is highly expressed in more than 85% of melanomas [25], irrespective of disease stage. Anti-MCSP antibodies have been commonly used to isolate CMCs through positive immunomagnetic enrichment [8,25,27] as well as for identification of CMCs by flow cytometry [26,28]. Expression of melanoma cell adhesion molecule (MCAM) is associated with an aggressive, invasive phenotype and upregulation is linked with disease progression [29-31]. Therefore, anti-MCAM might allow the isolation of CMCs exhibiting an aggressive phenotype which may have greater metastatic potential.

There has been recent discussion regarding the presence of stem-like tumour initiating cells in melanoma. Some research has shown that these cells may be responsible for tumour maintenance and renewal and may account for the fact that melanoma is notoriously difficult to treat. ATP-binding cassette sub-family B member 5 (ABCB5) and CD271 have been shown to be associated with a melanoma initiating cell phenotype. ABCB5 has been shown to be expressed in a subset of melanoma cells and is associated with tumour formation, metastasis and resistance to treatment [32-35]. CD271 expression has similarly been associated with increased capability to initiate tumour formation and metastasise [36]. ABCB5 and CD271 may be useful markers for targeting tumour initiating CMCs.

Here we conducted a pilot study to enrich and quantify CMCs from patient blood by targeting the MCSP, MCAM, ABCB5 and CD271 antigens with antibody coupled immunomagnetic beads. We evaluated the sensitivity of CMC isolation by enrichment with each individual bead type versus a combination of beads and compared the number of CMCs enriched between early stage, non-metastatic, and late stage, metastatic, melanoma patients.

## Methods

### Cell lines and antibodies

A2058 metastatic melanoma cells and human embryonic kidney 293 (HEK293) cells were cultured in DMEM supplemented with 10% foetal bovine serum (FBS).

Mouse anti-human monoclonal antibodies to MCAM (clone P1H12), MCSP (clone 9.2.27), CD271 (clone C40-1457) and CD45 (clone HI30) were purchased from BD Biosciences (San Jose, CA, USA). Rabbit anti-human polyclonal antibody to ABCB5 (clone RB16781) was purchased from Abgent (San Diego, CA, USA). Secondary antibodies were anti-mouse and anti-rabbit conjugated to Alexa-Fluor 488 (A488) and biotinylated anti-mouse, which was used with streptavidin-A488.

### Patient blood samples

Patients, recruited from 3 clinics in Perth, Australia, were diagnosed and staged according to guidelines of the American Joint Committee on Cancer (AJCC). All patients recruited between August 2011 and March 2012 were included in the study. Peripheral blood samples were obtained from 10 non-metastatic (stage I and II) and 13 metastatic (stage III and IV) patients. Additionally, 15 control blood samples were obtained from healthy volunteers. Blood was drawn by phlebotomists into EDTA tubes, after the first few millilitres was discarded to avoid epithelial contamination, and refrigerated until use. For 21 of 23 patients, blood was collected into five EDTA tubes; only one tube was collected for the remaining two patients and all controls. Patient samples ranged from 4 to 10 ml and control samples were 9 ml. All results were therefore calculated per ml of whole blood. Participants signed informed consent with the clinician in accordance with protocols safeguarding patient rights. All procedures have been accepted by the Human Research Ethics Committees at ECU (No. 2932) and SCGH (No. 2007–123).

### Immunofluorescence

Expression of MCAM, MCSP, ABCB5, CD271 and CD45 was tested in A2058 and HEK293 cells by immunofluorescence. Approximately 60,000 cells were seeded per coverslip and allowed to adhere overnight. Attached cells were fixed with 4% paraformaldehyde for 10 minutes at room temperature and stained with primary antibody, diluted in PBS with 3% BSA for one hour at room temperature. MCAM, MCSP and CD45 antibodies were diluted 1/500, ABCB5 1/250 and CD271 1/750. Cells were then stained with secondary antibody, diluted 1/500, for 20 minutes at room temperature. Cells were washed three times with PBS between each step. Coverslips were mounted onto microscope slides with ProLong Gold anti-fade mounting medium containing DAPI for nuclear staining (Invitrogen, Carlsbad, CA, USA) before analysis with an Olympus BX51 microscope equipped with an Olympus DP71 camera.

### Antibody-bead coupling

MCSP, MCAM, ABCB5 and CD271 antibodies were covalently bound to magnetic beads using the Dynabeads Antibody Coupling Kit (Invitrogen), as per the manufacturer’s instructions. For efficient antibody-bead coupling, 10 μg of antibody was used per mg of Dynabeads.

### Binding capacity of antibody coupled beads

A2058 cells were harvested in DMEM containing 5 mM EDTA and analysed with the Vi-Cell-XR Cell Viability Analyser (Beckman Coulter, Brea, CA, USA). 0.5 × 10^6^ A2058 cells were added to six tubes. 0.5 μl of MCSP beads was added to three tubes and 0.5 μl of MCAM beads was added to the remaining three tubes. Tubes were incubated for one hour at 4°C with rotation and subsequently placed on a DynaMag-2 magnet (Invitrogen). Enriched cells were washed three times with PBS, resuspended in 500 μl of PBS and counted with the Vi-Cell-XR Cell Viability Analyser. 0.5 μl of MCSP beads bound a mean of 1.42 × 10^5^ cells and 0.5 μl of MCAM beads bound a mean of 0.98 × 10^5^ cells.

### Quantification of spiked melanoma cells and patient CMCs

A2058 cells were harvested as previously described. Only cells with greater than 90% viability were used. Cells were spiked into DMEM/10% FBS or white blood cells (WBCs) resuspended in 1 ml incubation buffer (0.5% BSA, 2 mM EDTA in PBS, pH 7.2), following lysis of control blood with lysis buffer (140 mM NH4Cl, 17 mM Tris, pH 7.65). Spikes of 1, 10 and 20 cells were performed by pippetting under a microscope, while 50 and 100 cell spikes were performed by dilution. Cells were incubated with 0.5 μl of MCSP beads or a combination of MCSP, MCAM, ABCB5 and CD271 beads (with a combined volume of 0.5 μl) for one hour at 4°C with rotation. Enriched cells were placed on a DynaMag-2 magnet (Invitrogen), washed three times with PBS and mounted on microscope slides as previously described. Before mounting, cells enriched from control blood were fixed with 4% paraformaldehyde for 10 minutes at room temperature, stained with CD45 antibody (diluted 1/500 in PBS containing 3% BSA) for one hour at room temperature, followed by biotinylated anti-mouse and streptavidin-A488, both for 10 minutes at room temperature. Two PBS washes were performed between each antibody incubation. Enriched cells were quantified by microscopy where WBCs were defined as CD45 positive and melanoma cells as CD45 negative.

Control and patient blood was processed within 24 hours of collection. Blood was lysed as previously described. For controls and patients, one blood sample was incubated with a combination of MCSP, MCAM, ABCB5 and CD271 beads (combined volume 0.5 μl). The four remaining patient samples were incubated with 0.5 μl of one of the four individual bead types. Samples were incubated, washed and stained as previously described and quantified by microscopy.

### DNA amplification and detection of B-raf V600E mutation

Enriched cells attached to magnetic beads were lysed for whole genome amplification using the Repli-g kit (Qiagen, Germantown, MD, USA). In summary, after two PBS washes, all buffer was removed and cells attached to the magnetic beads were resuspended in 3.5 μl of cell lysis buffer containing DTT, incubated 10 minutes at room temperature then stop solution was added. Whole genome amplification was carried out as per the manufacturer’s instructions.

The V600E mutation was detected using the qBiomarker Somatic Mutation PCR Assay (c.1799 T > A) relative to a reference PCR for the B-raf gene (SABiosciences, Qiagen). Quantitative PCR was performed in a ViiA 7 Real-Time System (Applied Biosystems, Foster City, CA, USA).

### Statistics

Statistical tests were performed using SPSS Statistics 19 (IBM). The Mann–Whitney U Test was used to compare the number of CMCs between patients and controls and between metastatic and non-metastatic patients. The Wilcoxon Signed Rank test was used to compare the number of CMCs enriched with a combination of beads versus each individual bead type. P < 0.05 was considered statistically significant. The two patients with missing data were excluded from the appropriate analyses.

## Results

### Expression of markers in cell lines by immunofluorescence

**Figure 1:**
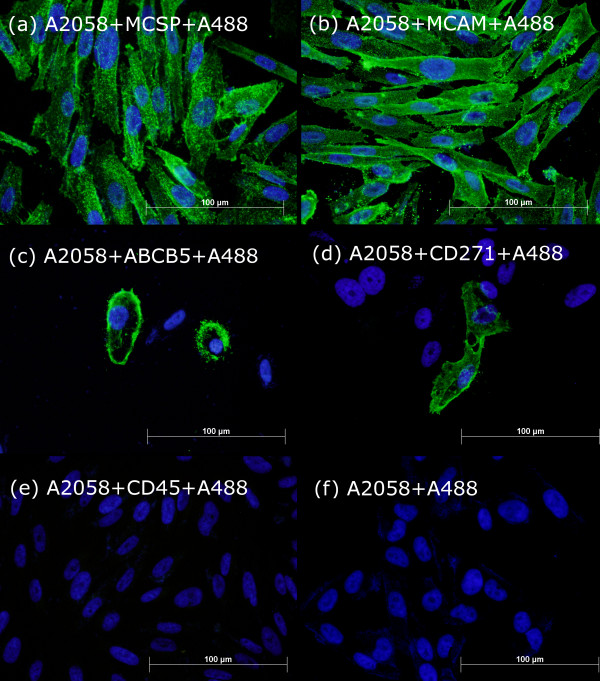
** Immunofluorescent analysis of A2058 melanoma cells.** A2058 cells were 100% positive for both MCSP (**a**) and MCAM (**b**). Less than 1% of cells were positive for both ABCB5 (**c**) and CD271 (**d**). All A2058 cells were CD45 negative (**e**). Anti-mouse A488-conjugate secondary antibody only staining was used as negative control and 100% of cells were negative (**f**)**.** All images are an overlay of blue (DAPI) and green fluorescence (specific marker), with the same exposure for green fluorescence. Original magnification 400x. Scale bars, 100 μm.

Expression of MCAM, MCSP, ABCB5, CD271 and CD45 was demonstrated in A2058 melanoma cells by immunofluorescence. HEK293 cells, used as negative controls, were 100% negative when stained with each antibody (not shown). A2058 cells were 100% positive for MCSP and MCAM (Figure 1a and Figure 1b respectively), while ABCB5 and CD271 were expressed on <1% of A2058 cells (Figure 1c and Figure 1d respectively), suggesting the presence of rare subpopulations within the cell line. A2058 cells were 100% negative when stained with CD45 (Figure 1e) and secondary antibody only (Figure 1f). This result supports the use of MCSP and MCAM to enrich the general CMC population. ABCB5 and CD271 appear useful for isolating rare CMC subsets.

### Sensitivity and specificity by retrieval of spiked A2058 cells

**Figure 2:**
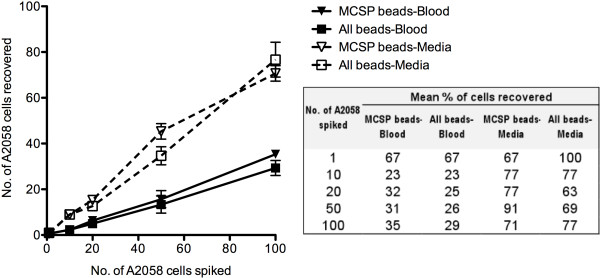
** Recovery of spiked A2058 cells from growth media and control blood with MCSP beads alone versus a combination of all beads.** The number of A2058 cells recovered from media (DMEM with 10% FBS) (dashed lines) was high compared to control blood (solid lines), as depicted in the graph. The average% recoveries of three independent spiking experiments are shown in the table.

To compare the sensitivity and specificity of enrichment targeting a single marker versus multiple markers (Figure 2) an increasing number of A2058 cells (1, 10, 20, 50 and 100) were spiked into media (DMEM/10% FBS) or control blood in triplicate and enriched with MCSP beads or a combination of all beads.

Percentage retrieval of cells spiked into media was high (mean = 76.7%). However, retrieval of cells from control blood was much less sensitive (mean = 35.8%), presumably due to the low specificity of the beads. This is supported by the observation of a large number of CD45 positive cells in the enriched fraction. Increasing the volume of beads increased the amount of non-specific binding but did not improve the recovery of spiked A2058 cells.

**Figure 3:**
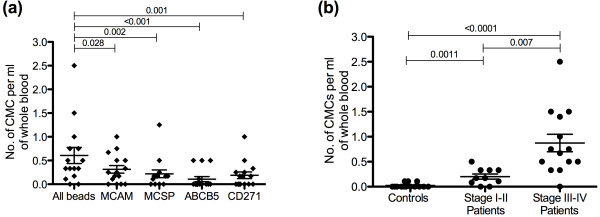
** Number of CMCs per ml whole blood in patients and controls.** (**a**) The number of CMCs per ml whole blood enriched from patients using MCAM, MCSP, ABCB5 and CD271 beads individually and in combination. (**b**) The number of CMCs per ml whole blood in healthy controls (n = 15), non-metastatic patients (stage I & II) (n = 10) and metastatic patients (stage III & IV) (n = 13) using a combination of all beads. The median and interquartile range of each group are indicated. The p-value of each comparison is denoted over the corresponding capped line.

The highest percentage recovery was observed in single cell spikes (Figure 2), however it should be noted that CD45 negative cells were counted as recovered A2058 cells in blood. As we occasionally observed single CD45 negative cells in control blood (Figure 3b), a CD45 negative cell enriched from a single cell spike may not always be an A2058 cell and may account for the increased percentage recovery of single A2058 cells from blood.

There was little difference between recoveries of spiked cells using MCSP beads alone versus a combination of beads. However, given our observation that 100% of A2058 cells are MCSP positive, the benefit of a combination of beads may not be apparent in the recovery of spiked cells as much as it may improve the recovery of CMCs from patient blood, where CMC phenotype is unknown.

In order to improve the sensitivity and specificity of the assay we tested the indirect method of immunomagnetic enrichment, where cells are first labelled with primary antibody and subsequently enriched with antibody-specific beads. We enriched spiked A2058 cells from control blood using the Dynabeads CELLection Pan Mouse IgG Kit (Invitrogen) according to the manufacturer’s instructions. While non-specific enrichment was decreased, the percentage of spiked cells recovered was also reduced (data not shown).

### Detection of CMCs in patient and control blood

**Figure 4:**
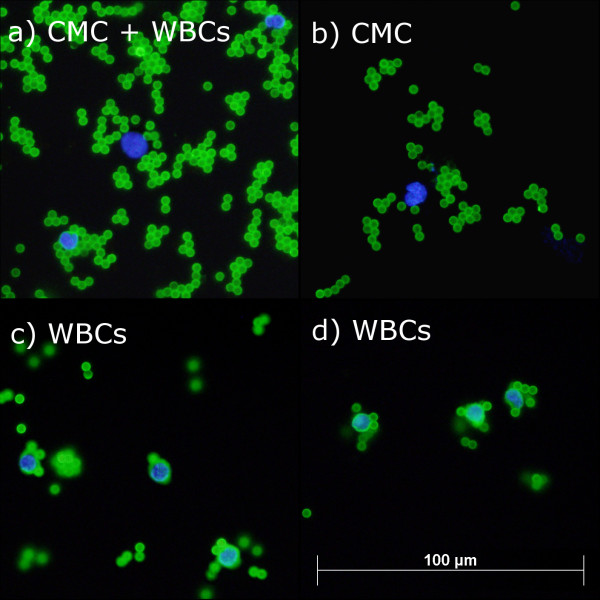
** CMCs enriched from patient blood.** (**a**) CMC (centre) and two CD45 positive WBCs, (**b**) CMC, (**c**) & (**d**) WBCs. The small green fluorescent spheres are the antibody-coupled beads.

CMCs were enriched from 15 control and 23 patient blood samples with a combination of MCSP, MCAM, ABCB5 and CD271 beads. In order to exclude non-specifically bound blood cells, enriched cells were stained for CD45, which is expressed on all human leukocytes [37]. Positively enriched CD45 negative cells were considered CMCs (Figure 4). A maximum of one CMC, or 0.11 CMCs per ml of whole blood, was detected in control blood samples. More than one CMC was detected in 73.9% of patients and ranged from 0 to 2.5 CMCs per ml (Figure 3b). There were significantly more CMCs per ml of whole blood in patients than controls (p < 0.001, Mann–Whitney U-Test). It is important to note that a maximum of one CMC was detected in 9 ml of blood in 4 of the 15 control samples. By contrast, increased numbers of CMCs were detected in patient samples even when lower volumes, usually 4 or 6 ml, were analysed. 

Although positive enrichment has been shown to result in higher purity than negative enrichment [26], we still observed low purity of CMCs (data not shown). We trialled a CD45 depletion step before positive enrichment to increase the purity of enriched CMCs, however, our results showed that CD45 depletion did not reduce the number of non-specifically enriched leukocytes (data not shown).

### Detection of V600E mutation in CMC enriched cells

An increasing number of A2058 cells (1, 5, 10 and 100) were spiked into control blood. Unspiked blood was used as a negative control in all experiments. Enriched cells attached to the beads were lysed and whole genome amplified. Amplified DNA was used to detect the B-raf V600E mutation by real-time PCR. A2058 cells are heterozygous for the V600E mutation [38]. We were consistently able to detect the mutation when 10 and 100 cells were spiked. However, PCR positivity was only observed in 30% of cases when 1 or 5 cells were spiked. This is in line with the percentage retrieval demonstrated above (Figure 2). It is possible that allele drop-out, which is a common problem in single-cell PCR [39], might have occurred in the samples where only one cell was present, reducing the number of tests positive for the B-raf V600E mutation in 1 and 5 cell spikes. Nevertheless, our results demonstrated that we were able to detect the V600E mutation in one cell amid a background of non-specifically bound leukocytes. The V600E mutation was further detected in CMCs enriched with a combination of magnetic beads in 6 of 8 patients tested, who had confirmed V600E-positive tumours. This result confirmed the enriched fraction contained tumour derived cells.

### Targeting a combination of markers versus a single marker for enrichment of CMCs

CMCs were enriched with antibody-coupled beads individually and in combination to see whether targeting multiple markers resulted in enrichment of more CMCs. Significantly more CMCs were enriched per ml whole blood when beads were used in combination rather than individually (MCAM, p = 0.028; MCSP, p = 0.002; ABCB5, p < 0.001; or CD271, p = 0.001; Wilcoxon Signed-Rank Test) (Figure 3a).

### CMCs in metastatic and non-metastatic patients

To determine whether there was a difference in the number of CMCs between early and late stage patients, CMCs were enriched in 10 non-metastatic and 13 metastatic patients. Significantly more CMCs were enriched from metastatic patients when a combination of beads was used (p = 0.007, Mann–Whitney U-Test) (Figure 3b). There was however no statistically significant difference when any individual marker was targeted.

## Discussion

Melanoma primary and metastatic tumours have highly heterogeneous expression patterns [20,22,27,40] and it is likely that CMCs also exhibit this heterogeneity. Thus here we undertook a novel strategy by targeting a combination of melanoma associated antigens and stem-cell markers to enrich CMCs from patient blood. Our results demonstrated that targeting multiple markers resulted in detection of more CMCs than targeting any individual marker tested. This suggests that the CMC population is heterogeneous and may explain why CMCs are detected in only a small percentage of patients in studies targeting a single marker. Ulmer et al. (2004) detected CMCs in 26% of melanoma patients across all stages when MCSP alone was targeted [8]. We propose the multi-marker approach allowed detection of cells with different phenotypes. Our finding is in line with research showing multi-marker RT-PCR assays are more sensitive than single marker assays [3,21,22,41]. Thus, different combinations of markers should be tested for optimal CMC enrichment. By targeting multiple cell surface markers the sensitivity of CMC detection was increased yet the specificity of the test was not reduced as no more than one CMC was detected in control blood.

Our spiking experiments showed immunomagnetic enrichment has low efficiency, as a consistently low percentage of spiked cells were recovered from blood. This result is similar to other studies trialling this technique [23,26] and suggests there is also low efficiency when enriching CMCs from patient blood. Thus, we infer that our assay allows us to detect only a proportion of the total number of CMCs in patient blood. However, comparison between groups is justified as a similar proportion of total CMCs should be detected in all samples.

In this study, CMCs were quantified by negative exclusion of CD45 positive cells, rather than by positive staining, as there are currently no markers known to be expressed on all CMCs [42]. Furthermore, we targeted tumour initiating CMCs that do not always express common melanoma associated antigens [36] so confirmation by positive staining of markers such as MART-1 or tyrosinase could result in exclusion of these cells. Positive staining for MCSP, MCAM, ABCB5 or CD271 was not feasible due to antigen binding sites being occupied by the antibody-coated beads.

CMCs were detected in all disease stages, suggesting cells are shed from primary tumours even early in disease progression. Some research suggests tumour cells can reach circulation during early stage disease, providing a population of dormant CMCs [43-45]. Since a larger number of CMCs were detected in metastatic patients than non-metastatic patients, quantification of CMCs may improve measures of disease progression. The presence of circulating tumour cells is associated with poor prognosis in early stage uveal melanoma [46] and other metastatic cancers [10-12,33,47]. Thus detection of CMCs in early stage patients may be useful for identifying those at risk of disease progression.

Here we showed the detection of the B-raf V600E mutation in the CMC enriched fraction. Similarly, previous studies have demonstrated the presence of this activating mutation in CMCs captured using anti-MCSP antibodies [25,27]. Furthermore, KRAS and EGFR mutations have been detected in circulating tumour cells from patients with metastatic colon and lung cancer respectively [48,49]. Moreover, serial genotyping of circulating lung cancer cells during gefitinib therapy demonstrated the emergence of the kinase inhibitor resistance–associated mutation T790M at increasing allelic ratios, coinciding with clinical relapse [48]. Genetic profiling of CMCs may prove valuable for monitoring the development of escape mutations during treatment. However, the extent to which CMCs represent the broader mutation profile of the parental tumours requires further investigation.

## Conclusion

In summary, the use of multiple markers to target CMCs provides a substantial improvement in sensitivity of isolation compared to previous studies targeting a single marker. Moreover, the use of multiple antibodies combined with negative exclusion of enriched cells allowed us to target rare cell populations. The detection of B-raf V600E confirms the presence of melanoma cells in the enriched fraction and supports the use of this methodology for genetic characterisation of CMCs. Our results indicate that quantification of CMCs may improve measures of disease progression and could further be used to evaluate treatment efficacy by determining whether CMCs are reduced following treatment. Additional research to evaluate the prognostic value of CMCs using this methodology is warranted where patients are monitored longitudinally to relate CMC numbers to disease outcome.

## Abbreviations

A488: Alexa-fluor 488; ABCB5: ATP-binding cassette sub-family B member 5; BSA: Bovine serum albumin; CD45: Cluster of differentiation 45; CD271: Cluster of differentiation 271; CMCs: Circulating melanoma cells; DAPI: 4',6-diamidino-2-phenylindole; DMEM: Dulbecco’s modified eagle medium; DTT: Dithiothreitol; EDTA: Ethylenediaminetetraacetic acid; EGFR: Epidermal growth factor receptor; FBS: Foetal bovine serum; HEK293: Human embryonic kidney 293; MCAM: Melanoma cell adhesion molecule; MCSP: Melanoma chondroitin sulfate proteoglycan; MART-1: Melanoma antigen recognized by T-cells 1; PBS: Phosphate buffered saline; PCR: Polymerase chain reaction; RT-PCR: Reverse transcription polymerase chain reaction; WBCs: White blood cells.

## Competing interests

The authors declare that they have no competing interests.

## Authors’ contributions

JBF developed the method of CMC enrichment and quantification and applied this to all control and patient blood samples. Immunocytochemistry, spiking experiments statistical analysis and drafting of the manuscript was also performed by JBF. ESG performed detection of the BRAF V600E mutation in enriched cells, helped draft the manuscript and critically revised the manuscript. MM recruited participants, provided patient blood samples and critically revised the manuscript. RP recruited participants, provided patient blood samples and critically revised the manuscript. MZ conceived of the study, participated in its design and coordination and critically revised the manuscript. All authors read and approved the final manuscript.

## Funding sources

Ziman, NHMRC Application number 1013349; Cancer and Palliative Care Research and Evaluation Unit WAPCN Small Grants 2010/11; and Cancer Council of WA Research Grant.
